# A functional role of LEFTY during progesterone therapy for endometrial carcinoma

**DOI:** 10.1186/s12964-017-0211-0

**Published:** 2017-12-21

**Authors:** Wu Fei, Daiki Kijima, Mami Hashimoto, Miki Hashimura, Yasuko Oguri, Sabine Kajita, Toshihide Matsumoto, Ako Yokoi, Makoto Saegusa

**Affiliations:** 1 Department of Pathology, Kitasato University School of Medicine, 1-15-1 Kitasato, Minami-ku, Sagamihara, Kanagawa 252-0374 Japan; 2 Department of Gynecology and Obstetrics, Jilin University Bethune Second Hospital, Changchun, People’s Republic of China

**Keywords:** Lefty, Endometrial carcinoma, Progesterone therapy, TGF-β, Smad, Cyclin a

## Abstract

**Background:**

The left-right determination factor (LEFTY) is a novel member of the TGF-β/Smad2 pathway and belongs to the premenstrual/menstrual repertoire in human endometrium, but little is known about its functional role in endometrial carcinomas (Em Cas). Herein, we focused on LEFTY expression and its association with progesterone therapy in Em Cas.

**Methods:**

Regulation and function of LEFTY, as well as its associated molecules including Smad2, ovarian hormone receptors, GSK-3β, and cell cycle-related factors, were assessed using clinical samples and cell lines of Em Cas.

**Results:**

In clinical samples, LEFTY expression was positively correlated with estrogen receptor-α, but not progesterone receptor (PR), status, and was inversely related to phosphorylated (p) Smad2, cyclin A2, and Ki-67 levels. During progesterone therapy, expression of LEFTY, pSmad2, and pGSK-3β showed stepwise increases, with significant correlations to morphological changes toward secretory features and decreased Ki-67 values. In Ishikawa cells, an Em Ca cell line that expresses PR, progesterone treatment reduced proliferation and induced increased expression of LEFTY and pGSK-3β, although *LEFTY* promoter regions were inhibited by transfection of PR. Moreover, inhibition of GSK-3β resulted in increased LEFTY expression through a decrease in its ubiquitinated form, suggesting posttranslational regulation of LEFTY protein via GSK-3β suppression in response to progesterone. In addition, overexpression or knockdown of LEFTY led to suppression or enhancement of Smad2-dependent cyclin A2 expression, respectively.

**Conclusion:**

Upregulation of LEFTY may serve as a useful clinical marker for the therapeutic effects of progesterone for Em Cas, leading to inhibition of tumor cell proliferation through alteration in Smad2-dependent transcription of *cyclin A2*.

**Electronic supplementary material:**

The online version of this article (10.1186/s12964-017-0211-0) contains supplementary material, which is available to authorized users.

## Background

Endometrial carcinoma (Em Ca) is the most common gynecologic malignancy. Although most patients are postmenopausal, approximately 5% of women with this disease are diagnosed before 40 years of age [1, 2]. This paradigm shift to younger ages may be due to an increase in the risk factors including marked obesity, diabetes mellitus, and hypertension [3, 4]. In these younger patients who want to have children in the future, hormone therapy using high-dose progesterone is frequently performed for the preservation of fertility [3–5].

Transforming growth factor β (TGF-β) is a member of the large family of structurally related cytokines that play an important role in controlling cell proliferation, differentiation, migration, and apoptosis [6–9]. Upon ligand binding, TGF-β receptor I recruits and subsequently induces an intracellular downstream signaling cascade involving Smad proteins which in turn act as transcription factors [6–9]. In human endometrium, TGF-β1 expression is up-regulated during periods of increasing plasma progesterone concentrations and down-regulated during its withdrawal, along with significant correlations to manifestation of various abnormalities including endometriosis, abnormal uterine bleeding, and Em Ca [10–15].

The left-right determination factor (LEFTY) is a novel member of the TGF-β superfamily, consisting of LEFTY1 and LEFTY2. They are both 366 amino acids long, and differ at only 16 residues, thus they are 97% identical and share 350 identical residues [16, 17]. LEFTY1/2 serve as repressors for TGF-β signaling by inhibiting the phosphorylation of Smad2 after activation of the TGF-β receptor [18]. LEFTY2, also known as endometrial bleeding-associated factor (ebaf), is controlled by ovarian steroids, and its expression correlates chronologically and spatially with that of bleeding-associated matrix metalloproteinases, indicating that LEFTY2 is a good candidate to locally modulate progesterone action on these proteinases within the endometrium [19–21].

In this study, we set out to first clarify the expression and the regulation of LEFTY1/2 in normal and malignant endometrium. Next, change in LEFTY1/2 expression in patients with Em Cas undergoing progesterone therapy was investigated. Finally, we examined the functional roles of LEFTY1/2 in TGF-β/Smad signaling pathways in Em Ca patients receiving the therapy.

## Methods

### Clinical cases

Histological findings were reviewed in hysterectomy specimens of endometrioid-type Em Cas from the case records of Kitasato University Hospital during the period from 2007 to 2015, according to the criteria of the 2014 World Health Organization classification. [22] Each case was also staged according to the 2009 International Federation of Gynecology and Obstetrics (FIGO) staging system. [23] A total of 120 cases of Em Cas, including 49 of grade (G)1, 14 of G2, and 57 of G3, as well as 22 samples of complex hyperplasia with or without atypia, were investigated. For the carcinoma cases, the mean age of the patients was 54.5 years (range from 30 to 84), and 53 were post-menopausal. In addition, our cases included 74 subcategorized as clinical FIGO stage I and 29 as stage II-IV, 27 with upper (≦1/2) myometrial invasion and 39 with lower (>1/2) myometrial invasion, as well as 18 that were positive for nodal metastasis and 67 that were negative.

A total of 32 patients with G1 Em Cas who received progesterone therapy were also selected (Table 1). These patients were diagnosed as stage I based on the findings of both physical and radiographic examinations, and their mean age was 30.3 years (range from 22 to 38). All patients received 400 to 800 mg of medroxyprogesterone acetate (MPA) via daily oral administration. Biopsy samples were taken by diagnostic curettage or hysteroscopy before and during progesterone therapy, and all contained sufficient tumor elements to allow examination of sequential morphological changes.

**Table 1:** Summary of Em Ca patients receiving progesterone therapy

						IHC markers			
Case No	Age (years)	When tissues are taken	Histology	MPA dosage/day	TE grade	LEFTY score	pSmad2 score	Ki-67 LI (%)	pGSK-3β score
1	29	before Tx	G1 Em Ca	^a^	^a^	4	6	30.6	4
		3 m	G1 Em Ca	600	3	6	8	18	*
		6 m	G1 Em Ca	600	4	12	12	4	4
2	30	before Tx	G1 Em Ca	^a^	^a^	6	4	2.2	6
		3 m	G1 Em Ca/AH	600	2	6	6	2.6	8
		6 m	G1 Em Ca/AH	600	3	12	8	1	^a^
		9 m	G1 Em Ca/AH	600	3	12	8	15.2	8
3	35	before Tx	G1 Em Ca	^a^	^a^	3	6	60.6	
		6 m	G1 Em Ca	600	2	3	6	22.2	8
4	35	before Tx	G1 Em Ca	^a^	^a^	3	9	26.4	4
		3 m	G1 Em Ca/AH	600	3	6	12	2	8
		9 m	G1 Em Ca/AH	600	3	12	12	11.4	6
5	34	before Tx	G1 Em Ca	^a^	^a^	0	6	3	8
		3 m	G1 Em Ca/AH	600	3	3	4	13	12
		before Tx	G1 Em Ca	^a^	^a^	9	3	60.5	4
		3 m	G1 Em Ca	600	4	12	6	8.4	6
6	35	before Tx	G1 Em Ca	^a^	^a^	0	8	19.8	4
		3 m	G1 Em Ca	600	3	0	8	17.6	8
		6 m	G1 Em Ca	600	4	3	6	6.2	8
7	29	before Tx	G1 Em Ca	^a^	^a^	4	8	50	6
		3 m	G1 Em Ca/AH	600	4	9	8	1	12
		9 m	G1 Em Ca/AH	600	4	12	12	5.5	12
8	37	before Tx	G1 Em Ca	^a^	^a^	6	12	20.2	6
		3 m	G1 Em Ca/AH	600	4	12	6	0	9
		6 m	G1 Em Ca	600	4	9	8	2.2	12
		9 m	G1 Em Ca/AH	600	4	8	4	3.5	12
9	24	before Tx	G1 Em Ca	^a^	^a^	9	8	33.4	9
		3 m	G1 Em Ca/AH	600	3	12	8	18.6	9
		6 m	G1 Em Ca/AH	600	3	12	12	48.2	9
10	29	before Tx	G1 Em Ca	^a^	^a^	4	4	52.5	9
		3 m	G1 Em Ca/AH	600	4	12	4	5	8
11	34	before Tx	G1 Em Ca	^a^	^a^	3	8	34.3	9
		3 m	G1 Em Ca/AH	600	4	6	8	2	12
		6 m	G1 Em Ca/AH	600	4	6	8	5	9
12	38	before Tx	G1 Em Ca	^a^	^a^	3	4	2	4
		3 m	G1 Em Ca/AH	800	4	6	6	1.3	8
		6 m	G1 Em Ca/AH	800	3	9	9	3	12
		9 m	G1 Em Ca/AH	800	4	12	6	0	12
13	30	before Tx	G1 Em Ca	^a^	^a^	4	9	34.5	6
		3 m	G1 Em Ca/AH	600	3	8	12	8.2	12
		6 m	G1 Em Ca	600	4	9	12	10.5	12
		9 m	G1 Em Ca/AH	600	4	8	12	20.7	12
14	29	before Tx	G1 Em Ca	^a^	^a^	3	8	55.8	8
		3 m	G1 Em Ca/AH	600	3	3	8	1	8
		6 m	G1 Em Ca/AH	600	3	4	9	2	12
15	22	before Tx	G1 Em Ca	^a^	^a^	3	4	60.8	6
		3 m	G1 Em Ca	600	1	6	8	35.4	9
		6 m	G1 Em Ca	600	3	6	8	13.5	12
16	38	before Tx	G1 Em Ca	^a^	^a^	4	8	18.8	8
		9 m	G1 Em Ca/AH	600	0	4	6	40.8	12
17	30	before Tx	G1 Em Ca	^a^	^a^	2	4	48.6	3
		3 m	G1 Em Ca	600	2	4	2	35.5	9
18	34	before Tx	G1 Em Ca	^a^	^a^	1	6	40	2
		3 m	G1 Em Ca	600	4	4	4	5.5	6
		6 m	G1 Em Ca	600	4	6	6	5	6
		9 m	G1 Em Ca/AH	600	4	4	9	4.6	8
19	26	before Tx	G1 Em Ca	^a^	^a^	4	9	17.8	
		3 m	G1 Em Ca	600	4	6	12	0	8
		6 m	G1 Em Ca	600	3	9	9	0	8
		9 m	G1 Em Ca/AH	600	4	9	9	9.6	12
20	28	before Tx	G1 Em Ca	^a^	^a^	4	6	30.5	6
		6 m	G1 Em Ca	600	3	6	4	7.6	12
21	30	before Tx	G1 Em Ca	^a^	^a^	2	4	52	3
		3 m	G1 Em Ca	400	3	9	9	6.5	9
22	32	before Tx	G1 Em Ca	^a^	^a^	6	8	43.6	9
		3 m	G1 Em Ca	400	4	8	4	20	12
		6 m	G1 Em Ca	600	4	12	12	3	8
23	28	before Tx	G1 Em Ca	^a^	^a^	6	12	43.5	8
		6 m	G1 Em Ca	600	4	9	12	8	12
24	25	before Tx	G1 Em Ca	^a^	^a^	1	8	18.8	4
		before Tx	G1 Em Ca	^a^	^a^	12	12	40.6	6
		before Tx	G1 Em Ca	^a^	^a^	6	6	67	8
		3 m	G1 Em Ca	400	4	9	9	7	8
		9 m	G1 Em Ca/AH	600	3	9	6	8.5	8
25	29	before Tx	G1 Em Ca	^a^	^a^	4	12	60.4	9
		before Tx	G1 Em Ca	^a^	^a^	4	4	14.2	4
		3 m	G1 Em Ca	400 mg/d	0	8	4	5	12
		6 m	G1 Em Ca	400	0	4	4	20.8	12
		9 m	G1 Em Ca	400	0	9	12	24	6
26	30	before Tx	G1 Em Ca	^a^	^a^	4	8	29.8	8
		3 m	G1 Em Ca	400	0	4	8	39	8
		6 m	G1 Em Ca	400	1	6	8	28.8	6
27	32	before	G1 Em Ca	^a^	^a^	9	9	18	9
		3 m	G1 Em Ca	400	2	8	8	3.3	12
28	29	before Tx	G1 Em Ca	^a^	^a^	6	6	15	6
		before Tx	G1 Em Ca	^a^	^a^	6	8	20	*
		3 m	G1 Em Ca	400	3	8	8	1	*
29	22	before Tx	G1 Em Ca	^a^	^a^	6	8	48	12
		3 m	G1 Em Ca	600	3	8	4	30.5	8
		3 m	G1 Em Ca	600	2	12	4	5.5	6
		6 m	G1 Em Ca	600	0	9	2	22.4	6
30	24	before Tx	G1 Em Ca	^a^	^a^	6	8	46	3
		3 m	G1 Em Ca	600	3	12	12	6	6
		6 m	G1 Em Ca	600	3	12	12	1	6
31	29	before Tx	G1 Em Ca	^a^	^a^	6	8	55.6	12
		3 m	G1 Em Ca	400	0	4	8	3.4	12
		9 m	G1 Em Ca/AH	400	2	9	8	7	12
32	30	before Tx	G1 Em Ca	^a^	^a^	3	8	63.8	3
		3 m	G1 Em Ca	600	0	8	8	18.5	8
		9 m	G1 Em Ca/AH	600	2	4	6	30.5	6

*TE* therapeutic efficacy, *Tx* therapy, *m* month, *G1 Em Ca* grade 1 endometrial carcinoma, *AH* atypical hyperplasia

^a^not examined

Ninety-six biopsy specimens of normal endometrial tissues including 24 in the proliferative phase, 52 in the secretory phase (10 early and 20 middle and late), and 20 in the menstrual phase were also investigated. All tissues were routinely fixed in 10% formalin and processed for embedding in paraffin. In addition, 40 fresh Em Ca samples (20 G1, 7 G2, and 13 G3), as well as 22 normal endometrial tissues were applied.

Histopathological analysis of endometrial tumors during progesterone therapy.

Evaluation of morphological changes that occurred during progesterone therapy was performed in accordance with methods described previously [24, 25]. Briefly, the sections obtained from tumors were examined in terms of the following four parameters: 1) cellularity, 2) nuclear rearrangement, 3) eosinophilia in the cytoplasm, and 4) the nuclear / cytoplasmic ratio. Therapeutic efficacy (TE) was graded by counting the numbers of altered parameters. Cases were subdivided into two categories, as follows: TE grade 0, 1, or 2 is defined as poor response; TE grade 3 or 4 is defined as good response.

### Antibodies and reagents

Anti-LEFTY, anti-Smad2, and anti-phospho(p)-Smad2 at serine 255 (pSmad2) antibodies were purchased from Abcam (Cambridge, MA, USA). Anti-p27^Kip1^ and anti-glycogen synthase kinase (GSK)-3β antibodies were bought from BD Biosciences (San Jose, CA, USA). Anti-p21^waf1^, anti-cyclin D1, and anti-Ki-67 antibodies were purchased from Dako (Copenhagen, Denmark). Anti-cyclin A2, anti-estrogen receptor α (ERα), and anti-progesterone receptor (PR) antibodies were from Novocastra (Newcastle, UK). Anti-pGSK-3β at Ser9 (pGSK-3β), anti-Akt, anti-pAkt at serine 473 (pAkt), and anti-ubiquitin antibodies were from Cell Signaling Technology (Danvers, MA, USA). Anti-β-actin antibody, nocodazole, 17β-estradiol (E2), medroxyprogesterone 17-acetate (MPA), MG132, and lithium chloride (LiCl) were purchased from Sigma-Aldrich Chemicals (St. Louis, MO, USA). Rapamycin and aphidicolin were obtained from Calbiochem (Cambridge, MA, USA). Recombinant TGF-β1 was purchased from R&D Systems (Minneapolis, MN, USA).

### Immunohistochemistry (IHC)

IHC was performed using a combination of microwave-oven heating and Histofine Simple Stain MAX-PO (MULTI) (Nichirei Biosciences, Tokyo, Japan) methods. For evaluation of IHC findings, scoring of nuclear and/or cytoplasmic immunoreactivity for LEFTY, pSmad2, ERα, and PR was performed as described previously [26, 27]. Briefly, the proportion of immunopositive cells among the total number of counted cells was subdivided into five categories as follows: 0, all negative; 1, <10%; 2, 10–30%; 3, 30–50%; and 4, >50% positive cells. The immunointensity was also subclassified into four groups: 0, negative; 1, weak; 2, moderate; and 3, strong immunointensity. IHC scores were generated by multiplication of the values of the two parameters. Nuclear immunopositivity for Ki-67 and cyclin A2 was also counted in at least 1000 cells in five randomly selected fields. Labeling indices (LIs) were then calculated as number per 100 cells, as described previously [26, 27].

### Plasmids and cell lines

The human *cyclin A2* promoter (GenBank accession number AF518006) between −1467 and +26 bp and the human *p27* ^*kip1*^ promoter (NG016341) encompassing −1565 to −12 bp (where +1 represents the transcription start site) were amplified by polymerase chain reaction (PCR) and were cloned into the pGL-3B vector (Promega, Madison WI, USA). Site-mutagenesis in putative GSK-3β phosphorylation motifs in the LEFTY1 protein (pcDNA3.1-LEFTY1 mutant type (mt) 5SA-HA) was also carried out using the PrimeSTAR Mutagenesis Basal kit (Takara Bio, Shiga, Japan) and pcDNA3.1-LEFTY-1wild-type (wt)-HA tag construct. The identity of all constructs was confirmed by sequencing prior to use. The sequences of PCR primers employed in this study are listed in Additional file 1: Table S1. pcDNA3.1-LEFTY1, pcDNA3.1-Smad2, pSG5-HEG0(ERα), pSG5-hPRB, pGL3B-(−3533) LEFTY1 luc, pGL3B-(−4805) LEFTY2 luc, pGL3B-(2338) p21^waf1^ luc, pGL3B-(−963) CD1 luc, and pSIREN-RetroQ- short hairpin (sh) LEFTY1 were used as described previously [26–29, 32].

Eight Em Ca cell lines, including Ishikawa, Hec1A, Hec6, Hec88, Hec108, Hec155, Hec180, and Hec251 cells, were used as described previously [30, 31]. Ovarian clear cell carcinoma cell line, ES-2, was obtained from the American Type Culture Collection (Manassas, VA, USA). ES-2 cells stably overexpressing LEFTY1 were also used as described previously [32]. In addition, ES-2 cells stably overexpressing LEFTY1wt-HA and LEFTY1mt 5SA-HA, and Ishikawa cells with knockdown of LEFTY1 by shRNA were also established.

### Transfection

Transfection was carried out using LipofectAMINE PLUS (Invitrogen, Carlsbad, CA, USA), in duplicate or triplicate, and luciferase activity was assayed as described previously [26, 27]. The siRNA against LEFTY1 or the negative control was transfected using the siPort NeoFx transfection agent (Ambion, Austin, TX, USA), according to the manufacturer’s instructions.

### RT-PCR (reverse transcription-PCR)

cDNA was synthesized from 2 μg of total RNA. Amplification by RT-PCR was carried out in the exponential phase to allow comparison among cDNAs synthesized from identical reactions using specific primers for LEFTY1/2 as described previously [32]. Primer sequences for both ERα and progesterone receptor B (PRB) are listed in Additional file 1: Table S1.

### Western blot assay and immunoprecipitation

Cells were lysed using RIPA buffer [50 mM/L, Tris-HCl (pH 7.2), 1% Nonidet P-40, 0.5% sodium deoxycholate, 0.1% sodium dodecyl sulfate] and clinical samples were lysed directly with 2× Laemmli sample buffer [65 mM/L Tris-HCl (pH 6.8), 5% 2-mercaptoethanol, 3% sodium dodecyl sulfate, 10% glycerol]. Aliquots of the proteins were resolved by SDS-PAGE, transferred to PVDF membranes, and probed with primary antibodies coupled to the ECL detection system (Amersham Pharmacia Biotechnology, Tokyo, Japan). The intensity of individual signals was measured using ImageJ software version 1.41 (NIH, Bethesda, MD, USA).

For immunoprecipitation, cells stably overexpressing LEFTY1 treated with LiCl and/or MG132 were lysed with TNE buffer [10 mM Tris-HCl (pH 7.6), 150 mM NaCl, 1% NP-40, 1 mM EDTA]. Cell lysates were cleared and incubated with anti-LEFTY antibody, followed by incubation with Protein G-Sepharose (Amersham Pharmacia Biotechnology). Western blot assay was subsequently performed with anti-LEFTY and anti-ubiquitin antibodies.

### Methylation analysis of the *LEFTY1* gene

The methylation status of the *LEFTY1* gene was analyzed as described previously [33]. Briefly, genomic DNA extracted from cell lines using Wizard Genomic DNA Purification kit (Promega) was treated with bisulfate using an EZ DNA Methylation-Gold kit (ZYMO Research, Orange, CA, USA). Bisulfate-treated DNA was amplified by PCR using specific primers as described previously. Amplicons were ligated into a pCR2.1 vector using the TA cloning kit (Invitrogen). Four positive clones were identified and sequenced from each sample. The DNA sequence generated was subjected to in silico analysis to describe the CpG methylation status of these clones.

### In situ hybridization (ISH)

Riboprobes for LEFTY1 and LEFTY2 were generated by in vitro transcription, and ISH assays were performed using the GenPoint Tyramide Signal Amplification System (Dako), as described previously [32, 34]. The ISH signal score was determined on the basis of the percentage of ISH signal-positive cells (1, less than 10% positive cells; 2, 10–30%; 3, 30–50%; 4, more than 50%) and the ISH signal intensity (0, none; 1, weak; 2, moderate; 3, strong) with the multiplication of the values of the two parameters.

### Flow cytometry

Cells were fixed using 70% ethanol and stained with propidum iodide (Sigma-Aldrich Chemicals) for cell cycle analysis. The prepared cells were analyzed by flow cytometry using BD FACS Calibur flow cytometer (BD Biosciences) and CellQuest Pro software (BD Biosciences).

### Statistics

Comparative data were analyzed using the Mann-Whitney *U*-test, chi-square test, and the Spearman’s correlation coefficient, whichever was appropriate. The cutoff for statistical significance was set as *p* < 0.05.

## Results

### LEFTY1/2 expression in normal and malignant endometrium

**Fig. 1:**
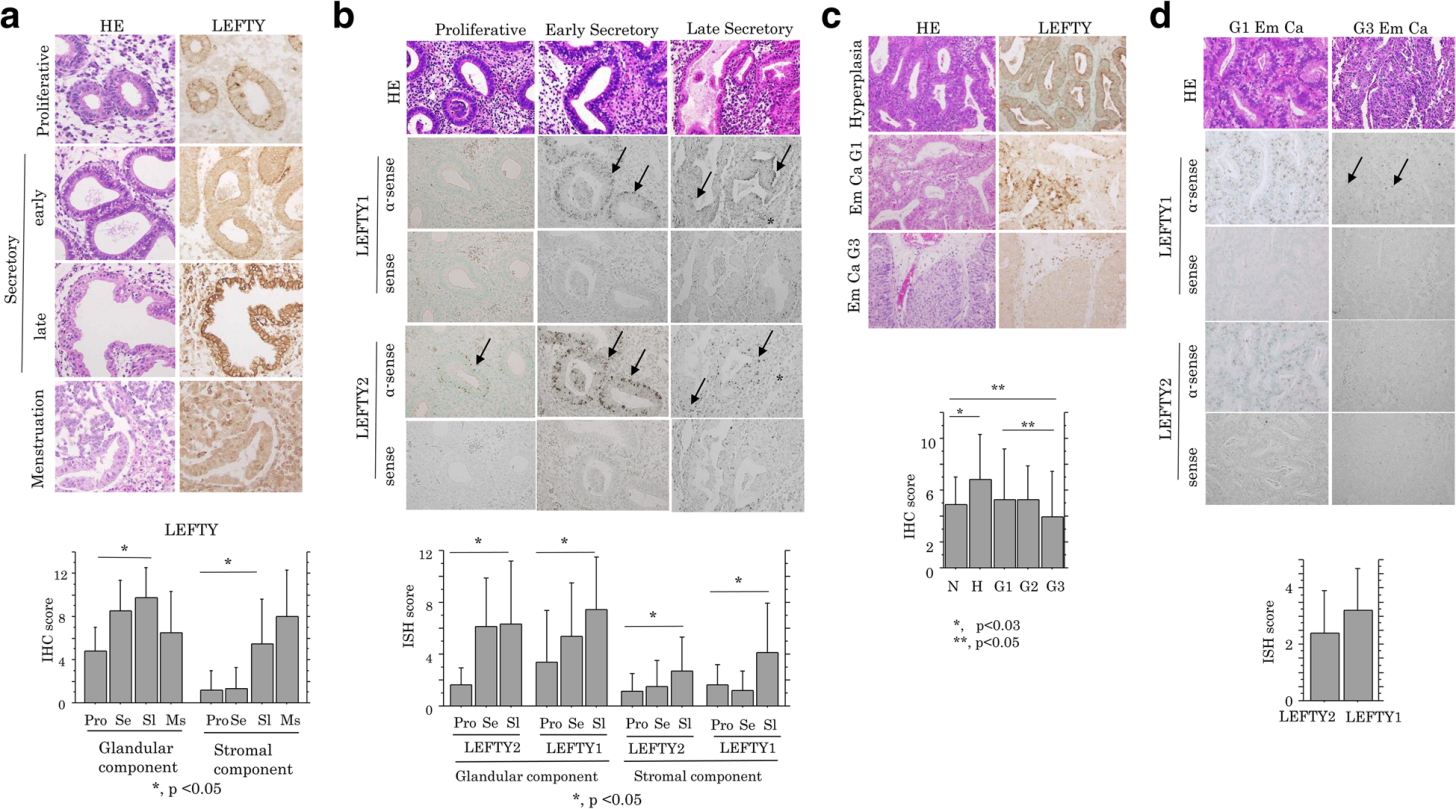
IHC and ISH findings for LEFTY expression in normal and malignant endometrium. **a** Upper: staining by hematoxylin and eosin (HE) and IHC for LEFTY during the menstrual cycle in normal endometrium. Original magnification, ×200. Lower: IHC score for LEFTY in glandular and stromal components of normal endometrium. **b** Upper: staining by HE and ISH for *LEFTY1* and *LEFTY2* mRNAs in normal endometrium. Note the ISH signals in glandular (indicated by arrows) and stroma components (indicated by asterisks). Original magnification, ×400. Lower: ISH scores for *LEFTY1* and *LEFTY2* mRNAs in glandular and stromal components of normal endometrium. **c** Upper: staining by HE and IHC for LEFTY in endometrial hyperplasia, and grade (G) 1 and G3 Em Cas. Original magnification, ×200. Lower: IHC score for LEFTY in normal (N), hyperplastic (H), and malignant (G1, G2, G3) endometrium. **d** Upper: staining by HE and ISH for *LEFTY1* and *LEFTY2* mRNAs in G1 Em Cas. Note the weak ISH signals for LEFTY1 mRNA in G3 Em Cas (indicated by arrows), in contrast to the strong signals in G1 tumors. Original magnification, ×400. Lower: ISH scores for *LEFTY1* (L1) and *LEFTY2* (L2) mRNAs in Em Cas. Pro, proliferative stage; Se, early secretory stage; Sl, late secretory stage; Ms., menstrual stage. All data shown are means ± SDs

Representative images of IHC findings for LEFTY during the menstrual cycle in normal endometrium are illustrated in Fig. 1a (upper panels). The anti-LEFTY antibody used in this study was able to react with both LEFTY1/2 as described previously [32]. Cytoplasmic LEFTY immunoreactivity was frequently observed in both glandular and stromal components. Average LEFTY score showed significant stepwise increase from the proliferative phase to late secretory or menstrual stage in both components (Fig. 1A, lower graph). Both *LEFTY1* and *LEFTY2* mRNA expression also showed similar cyclic changes, with no difference in the expression values between the two (Fig. 1b and Additional file 2: Figure S1A). In contrast, there were no differences in pSmad2 scores during the menstrual cycle (Additional file 2: Figure S1B).

Representative images of LEFTY immunoreaction in hyperplastic and malignant endometrium are also illustrated in Fig. 1c (upper panels). Average LEFTY score was significantly higher in hyperplastic lesions as compared to normal endometrium and showed significant decrease from hyperplasia through G1 to G3 Em Cas. The score was also significantly lower in G3 tumors as compared to normal lesions, but such associations were not evident between normal and either G1 or G2 tumor tissues (Fig. 1c, lower graph). No difference in the mRNA expression levels between *LEFTY1* and *LEFTY2* was also observed in Em Cas (Fig. 1d and Additional file 3: Figure S2A). Clinicopathologically, LEFTY score was significantly associated with advanced clinical stages and deep myometrial invasion, but not lymph node status (Additional file 3: Figure S2B).

Because active demethylation is essential for regulating a subset of TGF-β1-dependent genes [35], we further investigated changes in the CpG island status of the *LEFTY1* gene by sodium bisulfate sequencing. Although *LEFTY1* contained a region of CpG islands that was highly methylated in both Em Ca and normal tissues, the methylation status was not associated with LEFTY mRNA and protein expression (Additional file 4: Figure S3). These findings suggest that LEFTY1/2 were frequently expressed in normal and malignant endometrial tissues, independent of the gene methylation status.

### Associations of LEFTY with pSmad2 and ovarian hormone receptors in Em Cas

**Fig. 2:**
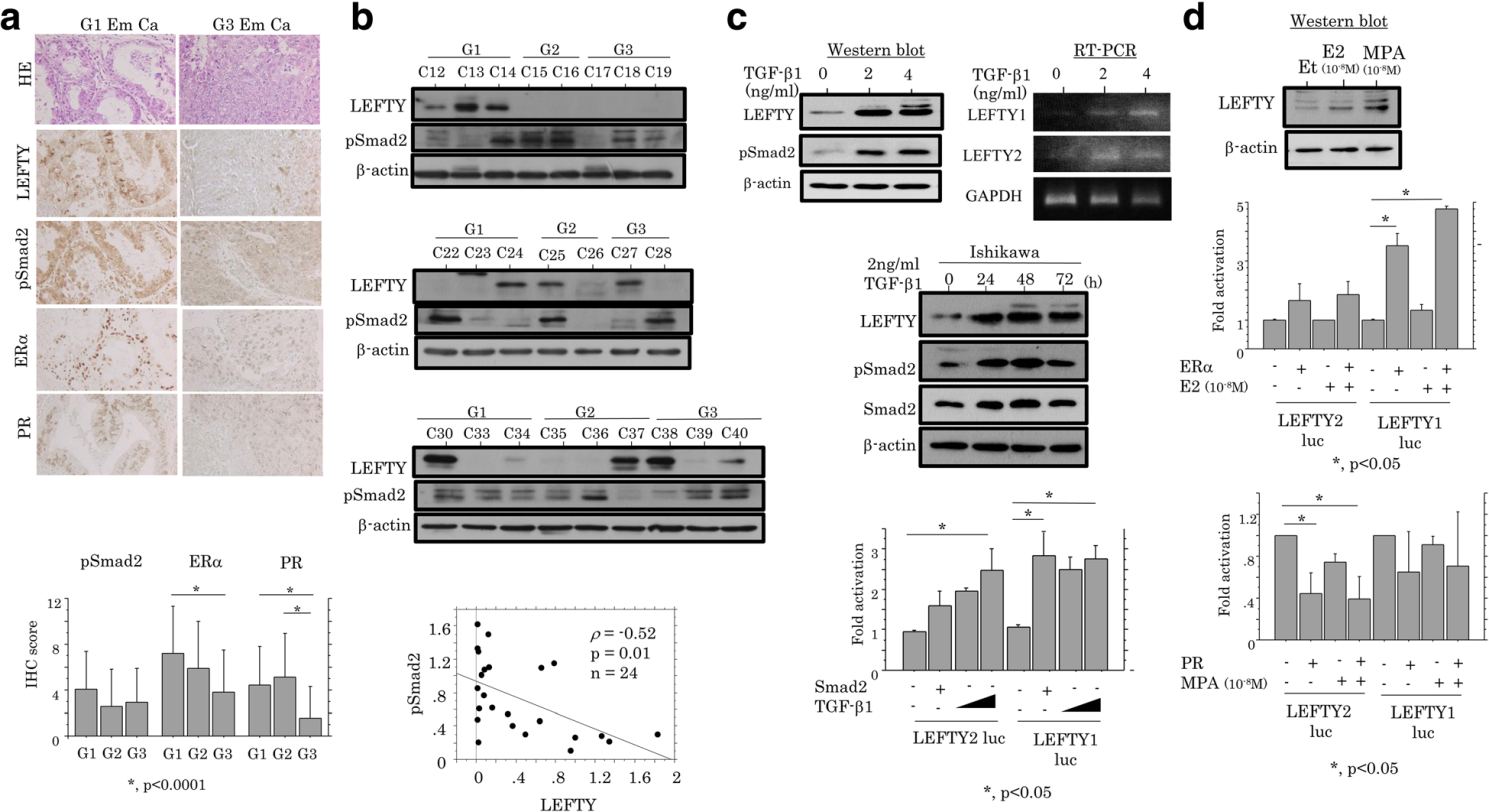
Associations of LEFTY with pSmad2 and ovarian hormone receptors in Em Cas. **a** Upper: staining by hematoxylin and eosin (HE) and IHC for LEFTY, pSmad2, ERα, and PR in Em Cas. Original magnification, ×200. Lower: IHC score for pSmad2, ERα, and PR in Em Cas. The data shown are means ± SDs. **b** Upper three panels: expression of LEFTY and pSmad2 proteins in Em Ca tissues by western blot assay. Lower: correlation between LEFTY and pSmad2 expression in Em Cas. Intensity of bands for endogenous LEFTY and pSmad2 proteins in upper three panels were calculated by normalization to β-actin using NIH Image software. *ρ*, Spearman’s correlation coefficient; p, *p*-value; N, number of cases. **c** Upper: western blot (left) and RT-PCR (right) analyses for the indicated molecules in Ishikawa cells following treatment with 0, 2, and 4 ng/mL TGF-β1 for 24 h. Middle: western blot analysis for the indicated proteins in Ishikawa cells following treatment with 2 ng/ml TGF-β1 for the times shown. Lower: Ishikawa cells were transfected with LEFTY1 and LEFTY2 reporter constructs, in addition to either cotransfection with Smad2 or TGF-β1 treatment. Relative activity was determined based on arbitrary light units of luciferase activity normalized to pRL-TK activity. The activities of the reporter plus the effector relative to that of the reporter plus empty vector are shown as means ± SDs. The experiment was performed in duplicate. **d** Upper: expression of LEFTY in Ishikawa cells with either 10^−8^ M 17β-estradiol (E2), 10^−8^ M medroxyprogesterone 17-acetate (MPA), or ethanol (Et) for 24 h. Middle and lower: Ishikawa cells were transfected with LEFTY1 and LEFTY2 reporter constructs, together with either ERα/E2 (middle) or PRB/MPA (lower). The experiment was performed in duplicate

Based on the above findings, we first investigated associations of LEFTY with the TGF-β/pSmad2 pathway and ovarian hormone receptor status in Em Ca tissues. Representative images of immunohistochemistry for LEFTY, pSmad2, ERα, and PR in Em Ca tissues are illustrated in Fig. 2a (upper panels). Nuclear/cytoplasmic immunoreactivity for pSmad2 was observed in Em Cas, independent of the histological malignancy, while distinct nuclear stainings for both ERα and PR showed significant stepwise decrease from G1 to G3 tumors (Fig. 2a, lower graph). The LEFTY score was positively correlated with ERα, but not PR and pSmad2, score (Table 2). In contrast, western blot analysis revealed an inverse association between LEFTY and pSmad2 expression (Fig. 2b and Additional file 5: Table S2).

**Table 2:** Correlations among IHC markers investigated in endometrial carcinomas

	LEFTY *ρ* (p)	pSmad2 *ρ* (p)	ERα *ρ* (p)	PR *ρ* (p)	Cyclin A2 *ρ* (p)
pSmad2	0.14	*	*	*	*
	(0.15)				
ERα	0.37	0.12	*	*	*
	(<0.0001)	(0.2)			
PR	0.3	0.16	0.61	*	*
	(0.002)	(0.08)	(<0.0001)		
Cyclin A2	−0.4	−0.19	−0.4	−0.44	*
	(<0.0001)	(0.05)	(<0.0001)	(<0.0001)	
Ki-67	−0.37	−0.24	−0.25	−0.22	0.75
	(<0.0001)	(0.01)	(0.0009)	(0.18)	(<0.0001)

*ρ* Spearman’s correlation coefficient, *IHC* immunohistochemistry, *, not examined

In Ishikawa cells, which express both ERα and PR (Additional file 6: Figure S4A), treatment with TGF-β1 resulted in increased LEFTY expression at both mRNA and protein levels, along with increased pSmad2 expression (Fig. 2c, upper panels). A time-course analysis of TGF-β1 treatment revealed that both pSmad2 and LEFTY expression increased within 48 h (h), and the former returned to basal levels after 72 h, in contrast to the continuous high expression of the latter (Fig. 2c, middle panels). Both *LEFTY1* and *LEFTY2* promoters were activated by either Smad2 transfection or TGF-β1 treatment (Fig. 2c, lower graph). LEFTY expression was also increased by either E2 or MPA treatment (Fig. 2d, upper panels). Both *LEFTY* promoters were activated by ERα transfection with or without E2 treatment, although we could not find any putative ERα-binding elements within both promoter regions (Fig. 2d, middle graph). In contrast, the promoter activities were suppressed by transfection of PRB, independent of MPA status (Fig. 2d, lower graph).

### Regulation of cell cycle progression by LEFTY in Em ca cells

**Fig. 3:**
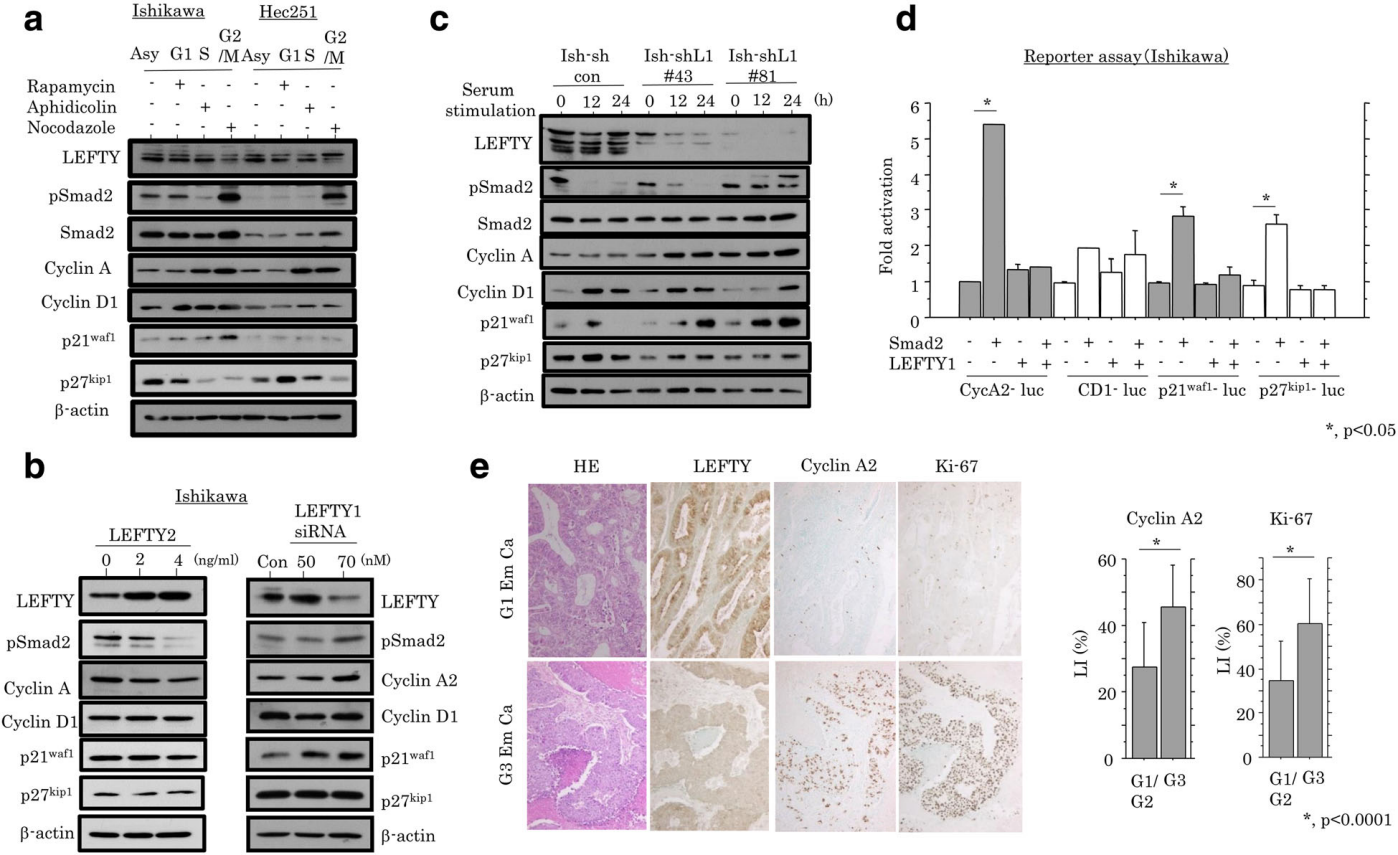
Regulation of cell cycle progression by LEFTY and pSmad2 in Em Cas. **a** Western blot assay for the indicated molecules in the Ishikawa and Hec251 cells, which were synchronized in the G1 phase by treatment with 50 nM rapamycin, in the early S phase with 2 μg/mL aphidicolin, or in the G2/M phase with 0.25 μg/mL nocodazole for 24 h. Asy, asynchronous. **b** Western blot assay for the indicated molecules in Ishikawa cells treated with 0, 2, and 4 ng/mL LEFTY2 for 24 h (left), and transfection of 50 and 70 nM LEFTY1 siRNA for 72 h (right). **c** Western blot analysis for the indicated proteins in Ish-shL1#43 and #81 stable cells and the mock cells for the times shown following restimulation with 10% serum after serum starvation for 24 h. **d** Ishikawa cells were transfected with cyclin A2, cyclin D1, p21^waf1^, and p27^kip1^ reporter constructs, in addition to cotransfection with Smad2 or LEFTY1. Relative activity was determined based on arbitrary light units of luciferase activity normalized to pRL-TK activity. The activities of the reporter plus the effector relative to that of the reporter plus empty vector are shown as means ± SDs. The experiment was performed in duplicate. (E) Left: staining by hematoxylin and eosin (HE) and IHC for LEFTY, cyclin A2, and Ki-67 in Em Cas. Original magnification, ×100. Right: labeling indices for cyclin A2 and Ki-67 in Em Cas. The data shown are means ± SDs

Since LEFTY2 can inhibit cell proliferation and suppress growth in some tumors [36], we examined an association between LEFTY expression and cell cycle progression using Ishikawa and Hec251 cells. In synchronized cells (Additional file 7: Figure S5A), LEFTY and p27^kip1^ expression was substantially decreased in G2/M-arrested cells, in contrast to increased expression of pSmad2, cyclin A2, and p21^waf1^ (Fig. 3a). Treatment of Ishikawa cells with recombinant LEFTY2 led to decreased expression of pSmad2, cyclin A2, and p21^waf1^, while LEFTY1 knockdown by siRNA resulted in increased expression of pSmad2, cyclin A2, and p21^waf1^ (Fig. 3b). To further examine an association between LEFTY1 and alterations in expression of several cell cycle-related molecules during cell growth, we established two independent Ishikawa cell lines with LEFTY expression blocked by a LEFTY1 specific shRNA. The cell lines were rendered quiescent by serum starvation and were subsequently stimulated with serum. At 12 and 24 h after release in the cell cycle, expression levels of pSmad2, cyclin A2, and p21^waf1^ were substantially increased relative to the mock cells, in contrast to the decreased p27^kip1^ expression (Fig. 3c). Transfection with Smad2 led to increased promoter activity of *cyclin A2*, as well as *p21* ^*waf1*^ and *p27* ^*kip1*^ genes, while these effects were abrogated by cotransfection with LEFTY1 (Fig. 3d, Additional file 7: Figure S5B). In clinical samples, both cyclin A2 and Ki-67 LI values were significantly higher in G3 Em Cas as compared to those of G1 tumors (Fig. 3e), in addition to an inverse correlation to LEFTY score (Table 2). These findings suggest that LEFTY participates in modulation of cell cycle progression through suppression of pSmad2-dependent transcription of the genes including *cyclin A2* in Em Cas.

### Changes in LEFTY expression during progesterone therapy for Em Cas

**Fig. 4:**
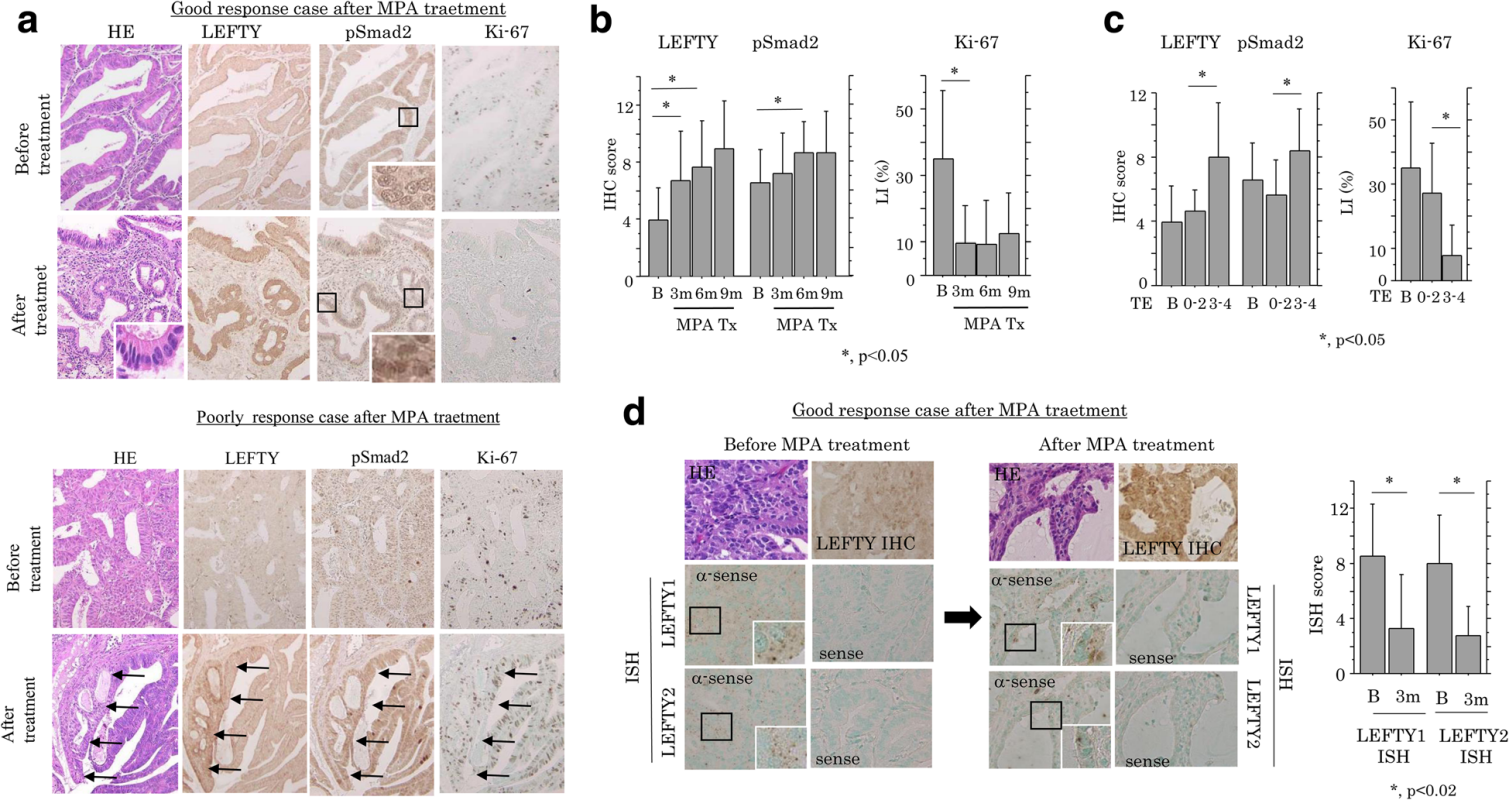
Changes in LEFTY expression during progesterone therapy for Em Cas. **a** Staining by hematoxylin and eosin (HE) and IHC for the indicated molecules in good (upper) and poor (lower) response cases after progesterone (MPA) treatment. Insets show the magnified views of the boxed areas in upper panels, while the focal carcinomatous areas with a weak response to the therapy are indicated by arrows in lower panels. Original magnification, ×100 and ×400 (inset). **b** IHC scores for LEFTY and pSmad2 (left) and Ki-67 LIs (right) in Em Ca cases before and after progesterone therapy (MPA Tx) for 3, 6, and 9 months (m). **c** IHC scores for LEFTY and pSmad2 (left) and Ki-67 LIs (right) in Em Ca cases before progesterone therapy and in therapeutic efficacy (TE) 0–2 and 3–4 groups. **d** Left and middle: staining by HE, IHC for LEFTY, and ISH for *LEFTY1* and *LEFTY2* mRNAs in Em Ca tissues before (left) and after MPA therapy (middle) in a good response case. Insets show the magnified views of the boxed areas. Original magnification, ×200 and ×400 (inset). Right: ISH score for *LEFTY1* and *LEFTY2* mRNAs in Em Ca cases before and after progesterone therapy for 3 months (m)

Since our data showed that LEFTY can be upregulated by E2 and MPA, we examined the impact of progesterone therapy on LEFTY expression using clinical Em Ca samples from patients receiving the therapy (Table 1). In the good response group (TE≧3), tumor cells showed marked changes in their morphological appearance, often becoming closer in resemblance to normal endometrial glandular components in the secretory phase, in contrast to the poor response group (TE≦2) (Fig. 4a). Average IHC scores for LEFTY and pSmad2 showed stepwise increases during progesterone therapy in Em Ca patients, which were significantly correlated with TE grading, while Ki-67 LIs were significantly decreased (Fig. 4b and c). In 10 cases selected from the good response group, *LEFTY1/2* mRNA expression was significantly decreased during the therapy, in contrast to the increased protein expression (Fig. 4d). In Ishikawa cells treated with MPA, there was a tendency towards a lower proliferation rate, along with increased expression of LEFTY and pSmad2, as well as p21^waf1^ and p27^kip1^ (Additional file 8: Figure S6). These findings suggest that upregulation of LEFTY and pSmad2 occurred during progesterone therapy in Em Cas, in line with changes in the morphology toward secretory features and lower cell proliferation.

### Association between LEFTY and GSK-3β during progesterone therapy for Em Cas

**Fig. 5:**
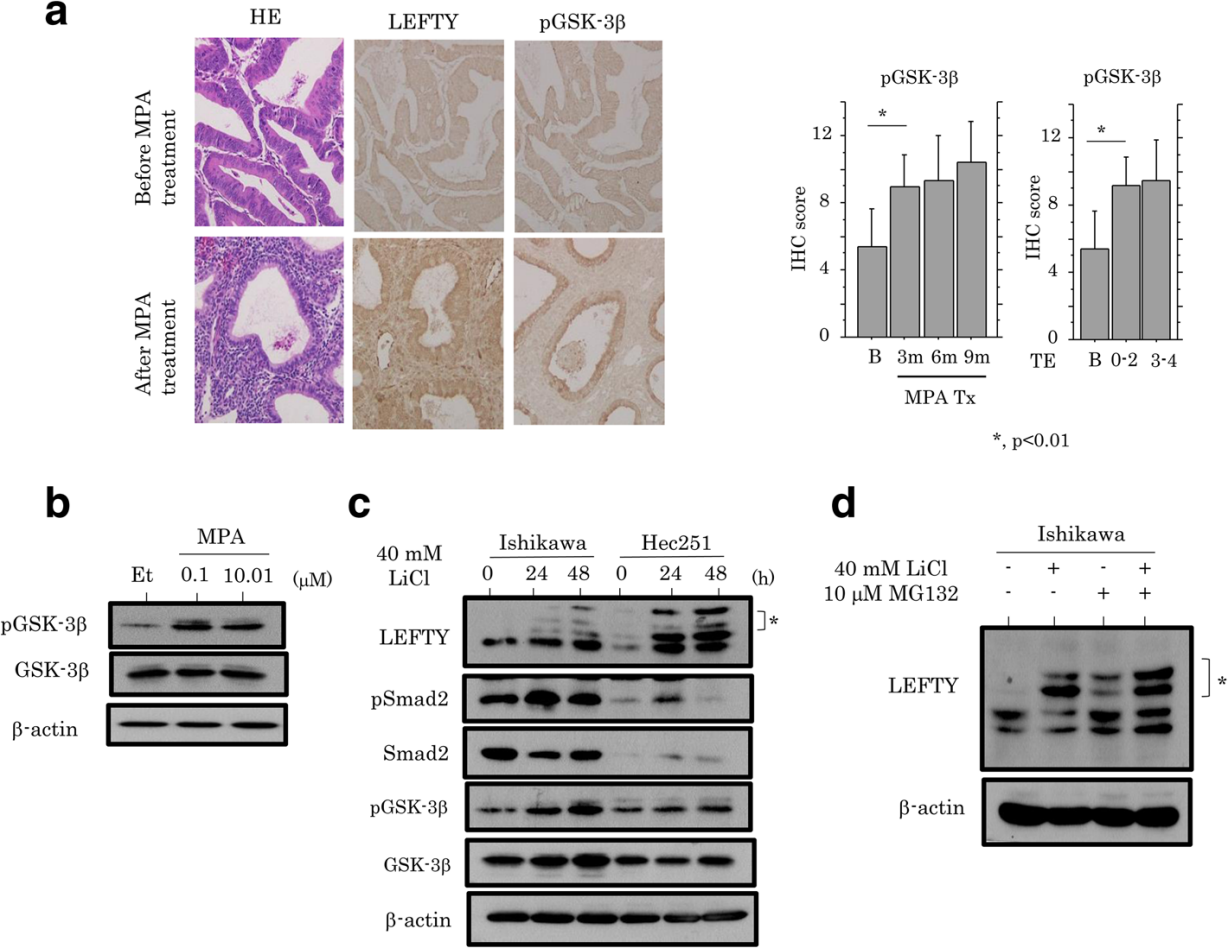
Association of LEFTY with GSK-3β during progesterone therapy for Em Cas. **a** Left: staining by hematoxylin and eosin (HE) and IHC for the indicated molecules in Em Ca cases with good response to progesterone therapy. Original magnification, ×200. Middle and right: IHC scores for pGSK-3β and its relation to therapeutic periods (middle) and therapeutic efficacy (TE) (right). B, before progesterone therapy; m, months. **b** Western blot analysis for the indicated molecules in Ishikawa cells treated with 10^−6^ or 10^−8^ M medroxyprogesterone 17-acetate (MPA) for 24 h. Et, ethanol. **c** Western blot analysis for the indicated molecules in Ishikawa and Hec251 cells following treatment with 40 mM LiCl for the times shown. Note the slower migrating bands of endogenous LEFTY (indicated by asterisk), probably due to phosphorylation of the protein. **d** Western blot analysis for LEFTY in Ishikawa cells following treatment with a combination of 40 mM LiCl for 24 h and 10 μM MG132 for 5 h. Note the slower migrating bands of endogenous LEFTY (indicated by asterisk)

Since GSK-3β is a major target of ovarian steroid hormones [37], we investigated changes in GSK-3β expression during progesterone therapy in Em Ca patients. The inactive pGSK-3β score was significantly increased during the therapy, independent of TE grading (Fig. 5a), in line with the findings from Ishikawa cells treated with MPA (Fig. 5b). Treatment of Ishikawa and Hec251 cells with LiCl, an inhibitor of GSK-3β revealed slower migrating bands of endogenous LEFTY, probably due to phosphorylation of the protein (Fig. 5c). To further examine whether LEFTY is tightly regulated by the ubiquitin-proteasome pathway, Ishikawa cells were also treated with a combination of LiCl and proteasome inhibitor, MG132. As shown in Fig. 5d, similar slow migration of LEFTY was also observed by treatment of MG132 with or without LiCl compared with those without the pretreatment.

**Fig. 6:**
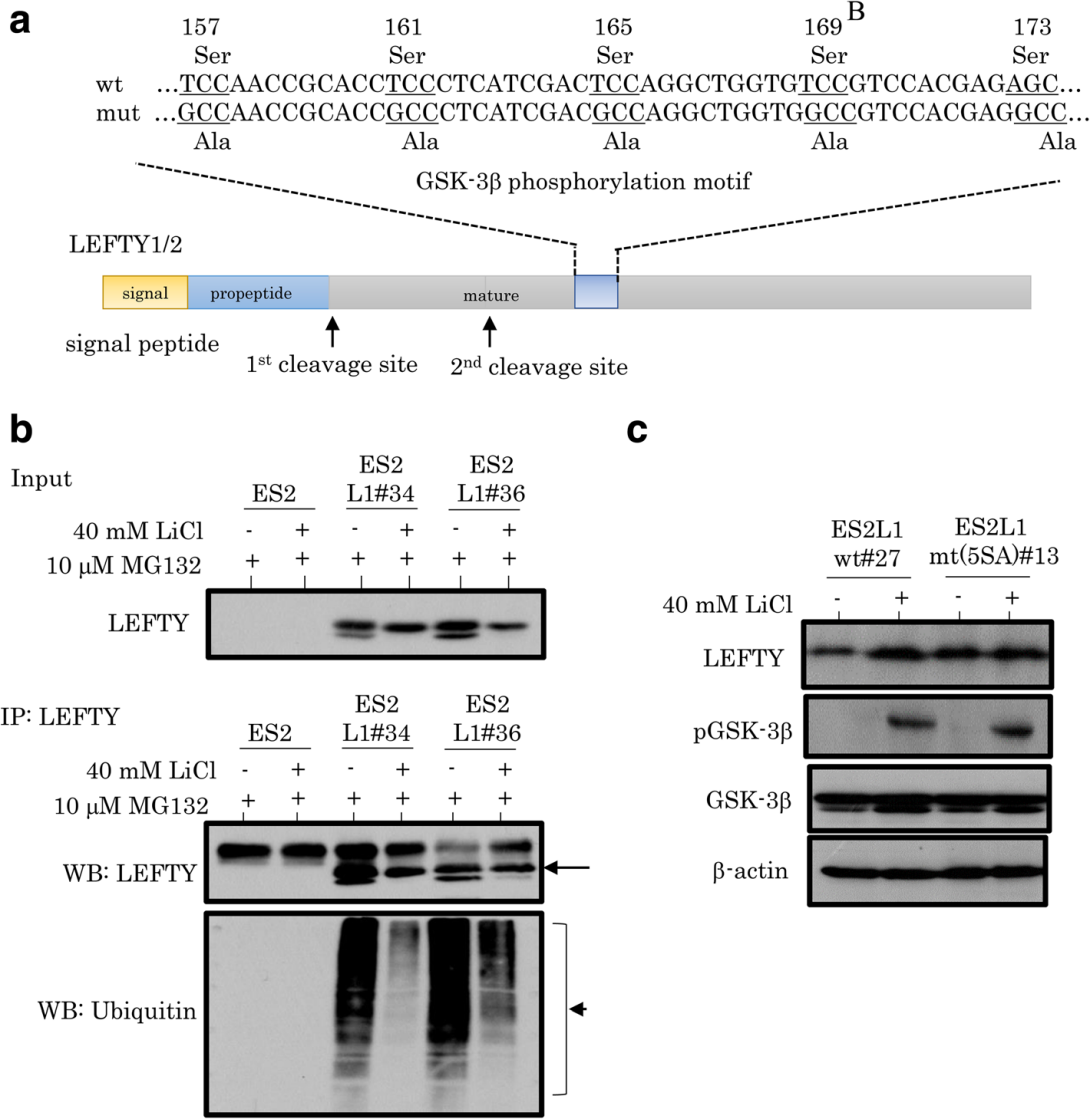
LEFTY is a GSK-3β substrate. **a** Structure of LEFTY1/2 protein showing the positions of five putative GSK-3β phosphorylation motifs. The corresponding constructs that were used in this study are also shown. wt, wild-type; mut, mutant type. **b** After treatment of ES-2 cells stably overexpressing LEFTY1 with 10 μM MG132 for 5 h with or without 40 mM LiCl for 24 h (upper: input), LEFTY (indicated by arrow) was immunoprecipitated (middle), and then ubiquitination of LEFTY (indicated by arrowhead) was examined (lower). **c** Western blot analysis for the indicated proteins after treatment of ES-2 cells stably overexpressing LEFTY1wt-HA or LEFTY1 mt (5SA)-HA with 40 mM LiCl for 24 h

Next, we noticed that LEFTY contains five putative GSK-3β phosphorylation motifs in the LEFTY1/2 protein (Fig. 6a). To examine whether LEFTY can be phosphorylated by GSK-3β and rapidly degraded through the ubiquitin-proteasome pathway, ES-2 cells stably overexpressing LEFTY1 were treated with LiCl and MG132, and LEFTY was immunoprecipitated. As shown in Fig. 6b, the level of ubiquitinated LEFTY1 was decreased by inhibition of GSK-3β activity following treatment with both LiCl and MG132, as compared to the treatment with MG132 alone. Changes in LEFTY expression by LiCl treatment were abrogated by mutations in the putative GSK-3β phosphorylation motifs (5SA) in the protein (Fig. 6c).

Finally, treatment of Ishikawa and Hec251 cells with TGF-β1 resulted in increased expression of pGSK-3β, as well as pAkt (Additional file 9: Figure S7). These findings suggest that the LEFTY protein is also posttranslationally regulated through alteration in GSK-3β activity in response to progesterone and TGF-β1.

## Discussion

The present study clearly provided evidence that LEFTY1/2 mRNA as well as protein showed a cyclic expression in both glandular and stromal components in normal endometrium, demonstrating that the expression was significantly higher in late secretory and menstrual stages. In contrast, it has been reported that the mRNA expression of *LEFTY2/ebaf* occurs primarily in stromal, but not epithelial, cells that have been predicidualized, indicating that it is a member of the premenstrual/menstrual molecular repertoire in human endometrium [19, 20]. One possible reason for the discrepant results may be the difference in sensitivity of mRNA detection. Given our previous study showing that LEFTY has biological effects in altering cell proliferation and cellular susceptibility to apoptosis in ovarian clear cell carcinoma [32], it appears that the molecule may also play an important role in the establishment and maintenance of homeostasis in normal endometrium through modulation of the terminal cell differentiation and the cell kinetics.

Although it has been reported that epigenetic factors participate in a mechanism underlying the regulation of LEFTY expression mediated by TGF-β1 [33], we found no association between *LEFTY1* gene methylation and its protein expression in normal and malignant endometrial tissues. In contrast, *LEFTY1/2* genes were transcriptionally upregulated by TGF-β1/Smad2 signaling in Ishikawa cells, while western blot analysis using Em Ca tissues revealed an inverse relation between LEFTY and pSmad2 expression. In addition, TGF-β1 treatment induced an increase in both pSmad2 and LEFTY expression within 48 h, and the former returned to basal levels after 72 h, in contrast to the continuous high expression of the latter. These findings allow us to speculate the existence of negative feedback loops among LEFTY, TGF-β, and Smad2, since LEFTY exerts significant inhibition of receptor-regulated Smad phosphorylation by TGF-β [18]. Moreover, TGF-β action has been demonstrated to be tightly controlled by a number of negative feedback mechanisms [38–40].

Interestingly, we found that LEFTY protein can be posttranslationally regulated by progesterone through alteration in GSK-3β activity in Em Cas from the following evidence: i) LEFTY protein contains five putative GSK-3β phosphorylation motifs and can be degraded through the ubiquitin-proteasome pathway; ii) inhibition of GSK-3β by LiCl resulted in increased expression of LEFTY, while the effects were abrogated by mutations in the five putative GSK-3β phosphorylation motifs in the protein; iii) pGSK-3β score was significantly increased during progesterone therapy in Em Ca tissues, in line with the findings demonstrating an increase in pGSK-3β expression by progesterone in Ishikawa cells; and iv) *LEFTY1/2* mRNA expression levels were significantly decreased during the therapy, probably through transcriptional repression of the promoter activities by progesterone signals, despite the increased LEFTY protein expression.

We also found that LEFTY was positively associated with ERα status in Em Ca tissues and *LEFTY1/2* expression was transcriptionally regulated by estrogen and ERα in Ishikawa cells. In addition, LEFTY expression was significantly higher in hyperplastic endometrial tissues as compared to those of both normal and malignant lesions, in line with the notion that endometrial hyperplasia is induced by continuous exposure to estrogen unopposed by progesterone [41]. However, it seems likely that the upregulation may be indirectly affected by estrogen-related signaling, since both *LEFTY1/2* promoter regions lack any putative ERα-binding elements. Whatever the case, these findings suggest that ovarian hormones may be major regulators of LEFTY expression at transcriptional and posttranslational levels in Em Cas.

Several lines of evidence from the present study support the conclusion that LEFTY expression is associated with lower cell proliferation in Em Cas, probably through suppression of Smad2-dependent cyclin A2 expression. First, the concordant upregulation of pSmad2 and cyclin A2 expression in the G2/M phase was correlated with decreased LEFTY expression during cell cycle progression. Second, overexpression of LEFTY2 or knockdown of LEFTY1 resulted in decreased or increased expression, respectively, of both pSmad2 and cyclin A2. Given that LEFTY1/2 are both 366 amino acids long, and they are 97% identical and share 350 identical residues [16, 17], it seems likely that both LEFTY1/2 may have similar effects on cell cycle progression. Further studies to investigate this are clearly warranted. Third, LEFTY inhibited Smad2-mediated promoter activation of *cyclin A*2. Finally, LEFTY score was negatively correlated with both cyclin A2 and Ki-67 LIs in Em Cas. Together with our results that demonstrated high LEFTY expression in Em Cas of advanced stages and with deep myometrial invasion, it appears that LEFTY may have anti-tumor effects through suppression of aggressive tumor growth by blocking TGF-β/Smad2 action in Em Cas. This conclusion is supported by the evidence showing a promotion of late-stage tumor progression, invasion, and metastasis by TGF-β and a reduction of tumor growth in nude mice by introduction of LEFTY to tumor cells [42–45].

An important finding in this study was that LEFTY expression was significantly increased during progesterone therapy for Em Cas, in line with changes in the morphology toward secretory features and the inhibition of cell proliferation. The pSmad2 score also showed a significant increase during the therapy, despite increased LEFTY expression. This may be due to an increase in secretion of TGF-β1 by progesterone [46], which in turn resulted in further activation of TGF-β1/Smad2-dependent transcription of *LEFTY1/2* genes. Moreover, treatment of Ishikawa cells with TGF-β induced an increase in signaling through the pAkt/pGSK-3β axis, which likely reflected LEFTY stability, indicating the existence of complex regulatory mechanisms for LEFTY expression during progesterone therapy in Em Cas.

## Conclusion

**Fig. 7:**
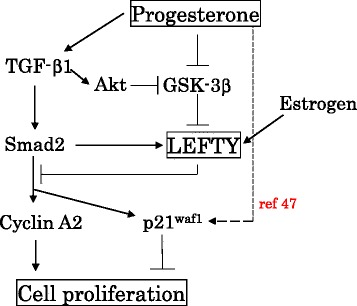
Schematic representation of the association of LEFTY with TGF-β/Smad2 signaling during progesterone therapy for Em Cas

Our observations suggest some novel functional roles of LEFTY in Em Ca cases receiving progesterone therapy (Fig. 7). Prolonged progesterone therapy induces upregulation of LEFTY through TGF-β-dependent or -independent GSK-3β inactivation. Estrogenic stimulation may be also due to transcriptional upregulation of LEFTY. The increased LEFTY expression leads to inhibition of cell proliferation, probably through changes in Smad2-mediated transcription of genes including *cyclin A2*, in line with changes in their morphological appearances toward secretory features. Activation of other signal pathways such as the β-catenin/p53/p21^waf1^ axis in response to progesterone therapy also contributes to the suppression of cell cycle progression [47]. Thus, LEFTY may serve as a useful clinical marker for the therapeutic effects of progesterone for Em Cas.

## Additional files

Additional file 1: Table S1. Primer sequences used in this study. (XLSX 10 kb)

Additional file 2: Figure S1. Expression of LEFTY1/2 mRNA and pSmad2 protein in normal endometrium. (TIFF 4603 kb)

Additional file 3: Figure S2. LEFTY1/2 mRNA expression and associations of LEFTY protein expression with clinicopathological factors in endometrial carcinomas. (TIFF 1138 kb)

Additional file 4: Figure S3. Relationship between *LEFTY1* methylation and its expression levels in normal and malignant endometrial tissues. (TIFF 3843 kb)

Additional file 5: Table S2. Relationship between LEFTY and pSmad2 expression as detected by western blot assay in endometrial carcinomas. (XLSX 9 kb)

Additional file 6: Figure S4. mRNA expression of ovarian hormone receptors in endometrial carcinoma cell lines. (TIFF 1467 kb)

Additional file 7: Figure S5. Cell cycle analysis and reporter assay for several cell cycle-related genes. (TIFF 786 kb)

Additional file 8: Figure S6. Changes in cell growth and expression of cell cycle-related molecules in Ishikawa cells in response to MPA treatment. (TIFF 1007 kb)

Additional file 9: Figure S7. Association between TGF-β and Akt/GSK-3β pathways in endometrial carcinoma cells. (TIFF 1083 kb)

## Abbreviations

E2: 17β-estradiol
Em Ca: endometrial carcinoma
ERα: estrogen receptor-α
GSK-3β: glycogen synthase kinase-3β
IHC: immunohistochemistry
ISH: in situ hybridization
LEFTY: left-right determination factor
LiCl: lithium chloride
MPA: medroxyprogesterone 17-acetate
PR: progesterone receptor
PRB: progesterone receptor B
TGF-β1: transforming growth factor-β1
