# Supplementary figures and images for: A functional role of LEFTY during progesterone therapy for endometrial carcinoma
Source: Cell Commun Signal. 2017 Dec 21;15:56. doi: 10.1186/s12964-017-0211-0 (PMC5740891; doi:10.1186/s12964-017-0211-0)

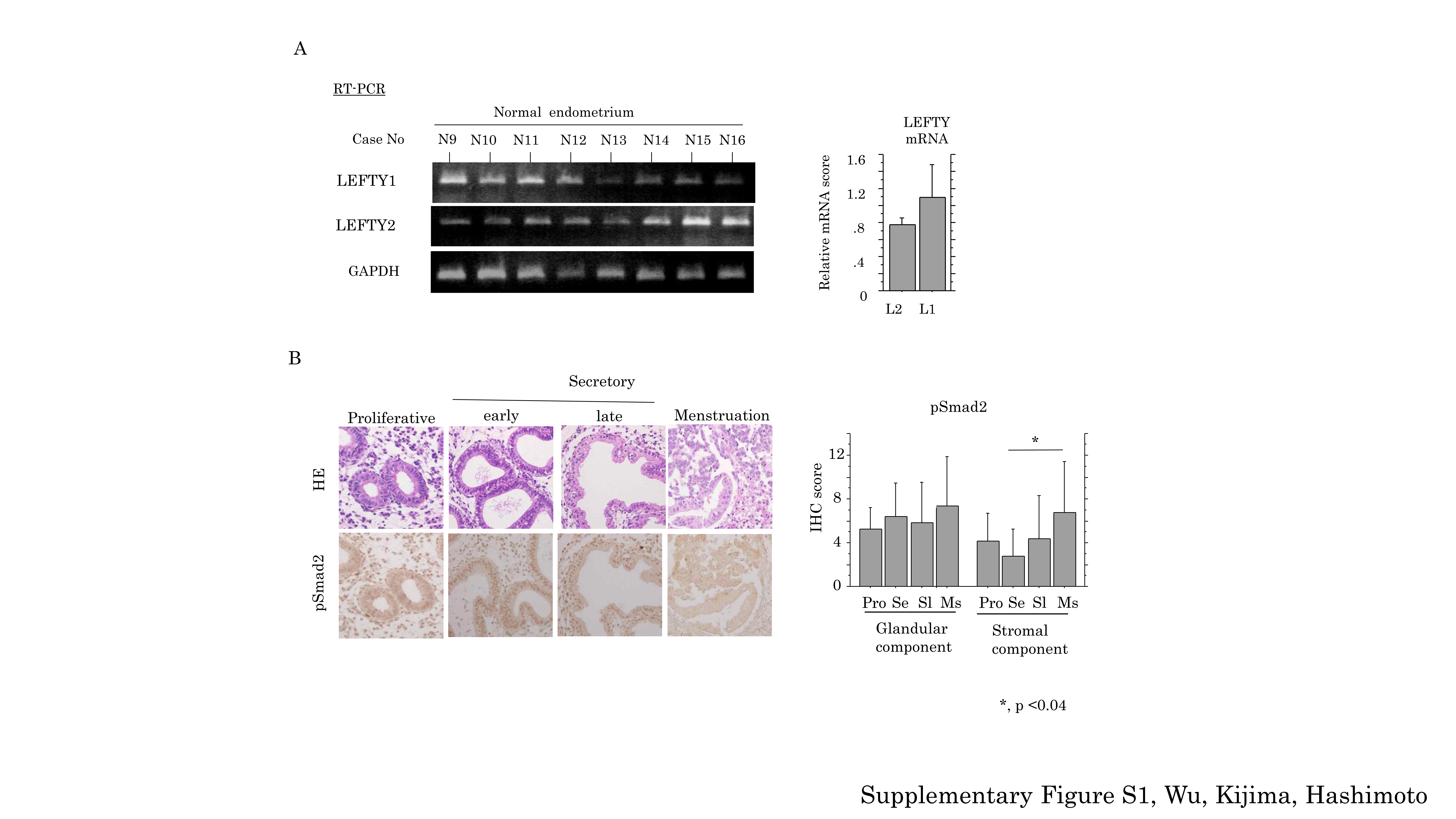

Supplement: Supplementary file 2 — Expression of LEFTY1/2 mRNA and pSmad2 protein in normal endometrium. (TIFF 4603 kb) [file 12964_2017_211_MOESM2_ESM.tif]

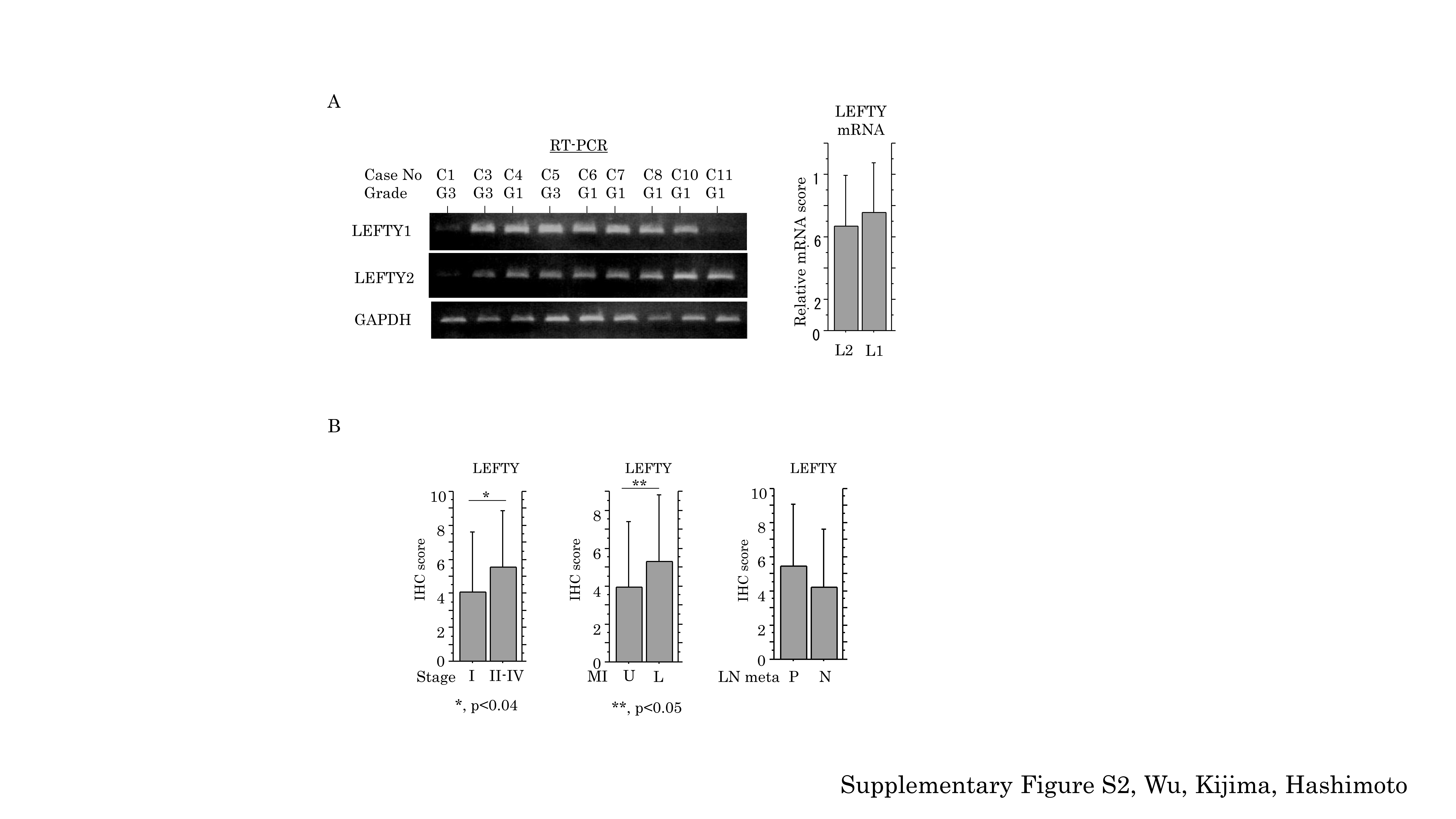

Supplement: Supplementary file 3 — LEFTY1/2 mRNA expression and associations of LEFTY protein expression with clinicopathological factors in endometrial carcinomas. (TIFF 1138 kb) [file 12964_2017_211_MOESM3_ESM.tif]

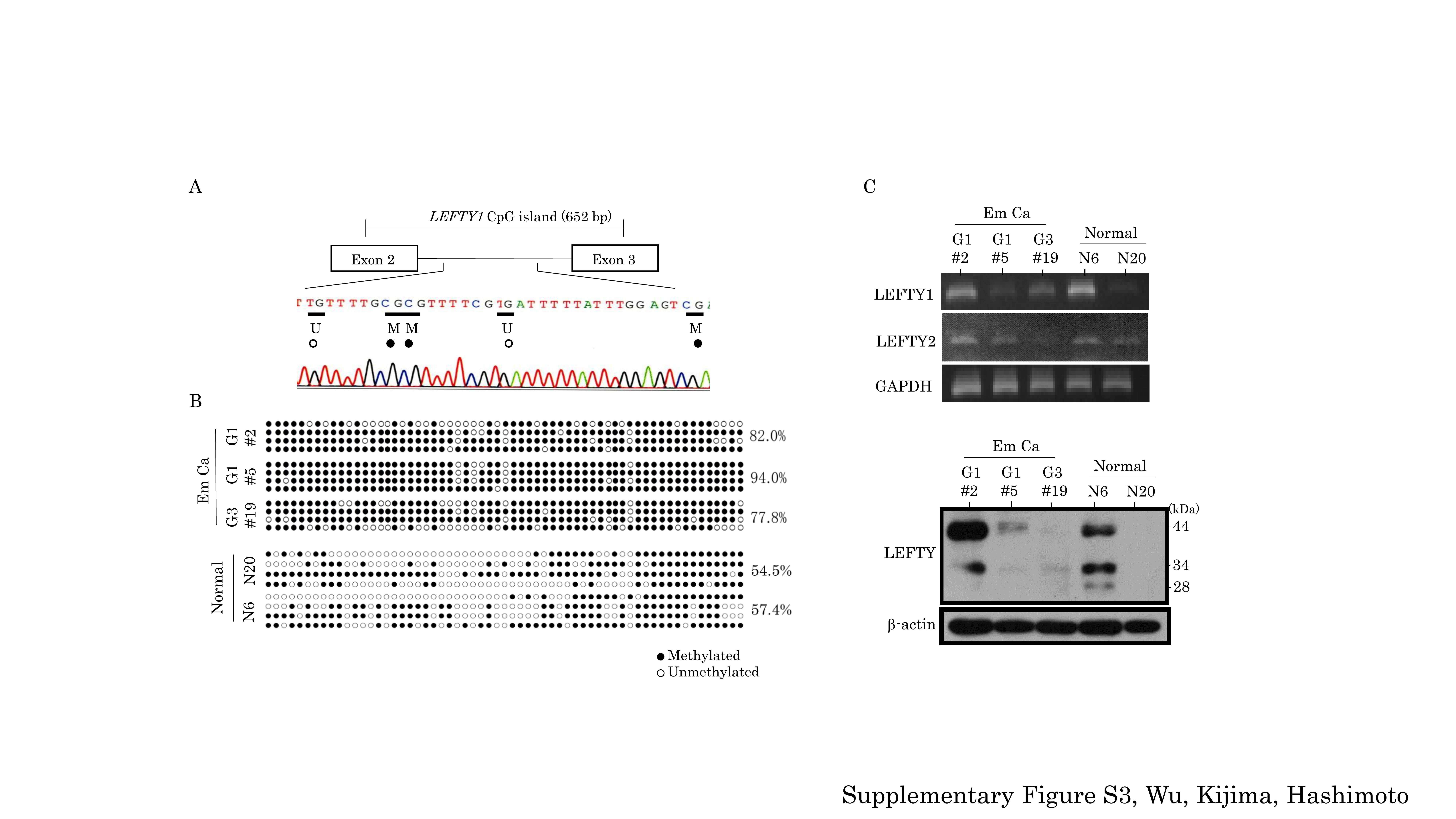

Supplement: Supplementary file 4 — Relationship between LEFTY1 methylation and its expression levels in normal and malignant endometrial tissues. (TIFF 3843 kb) [file 12964_2017_211_MOESM4_ESM.tif]

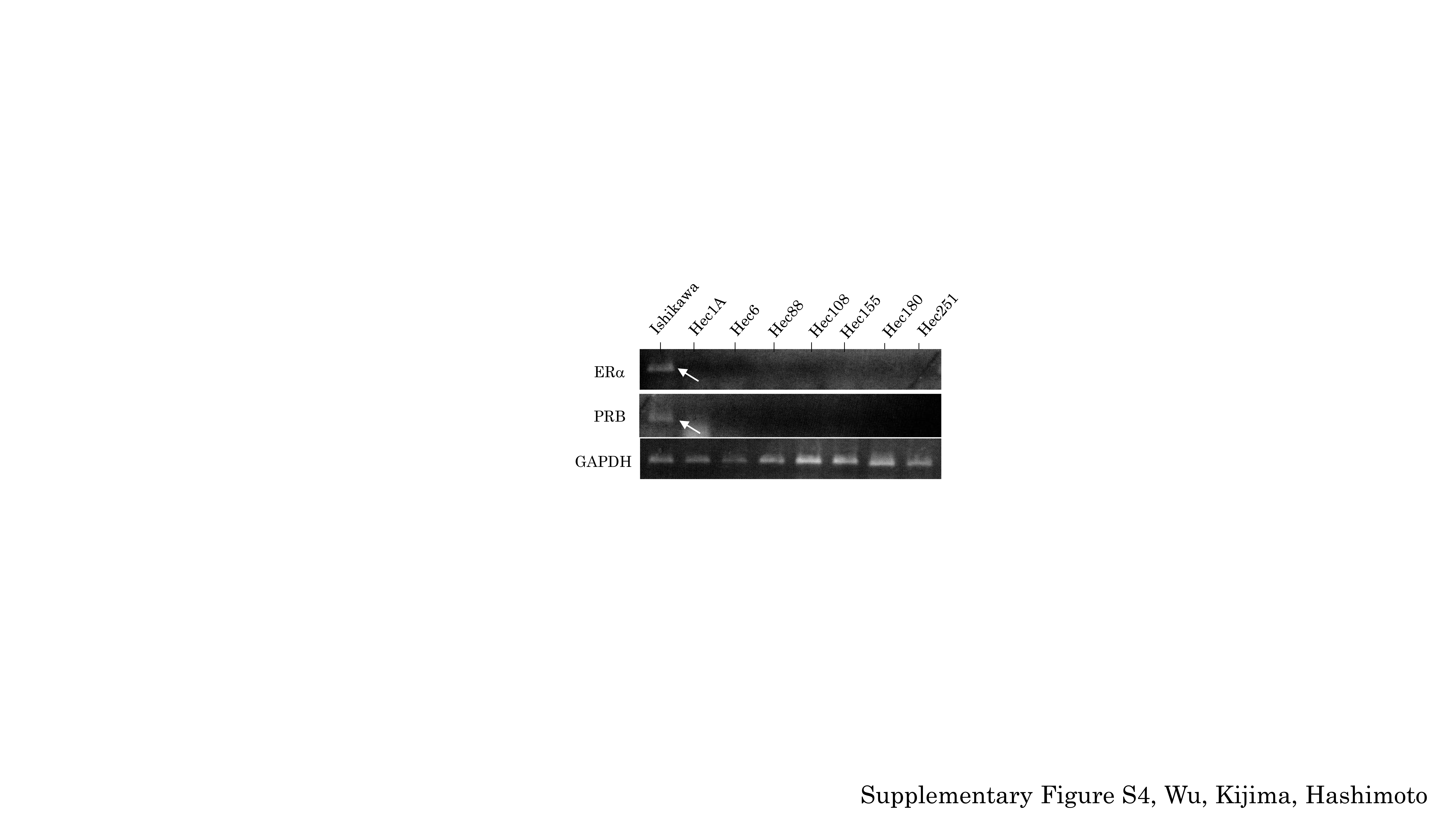

Supplement: Supplementary file 6 — mRNA expression of ovarian hormone receptors in endometrial carcinoma cell lines. (TIFF 1467 kb) [file 12964_2017_211_MOESM6_ESM.tif]

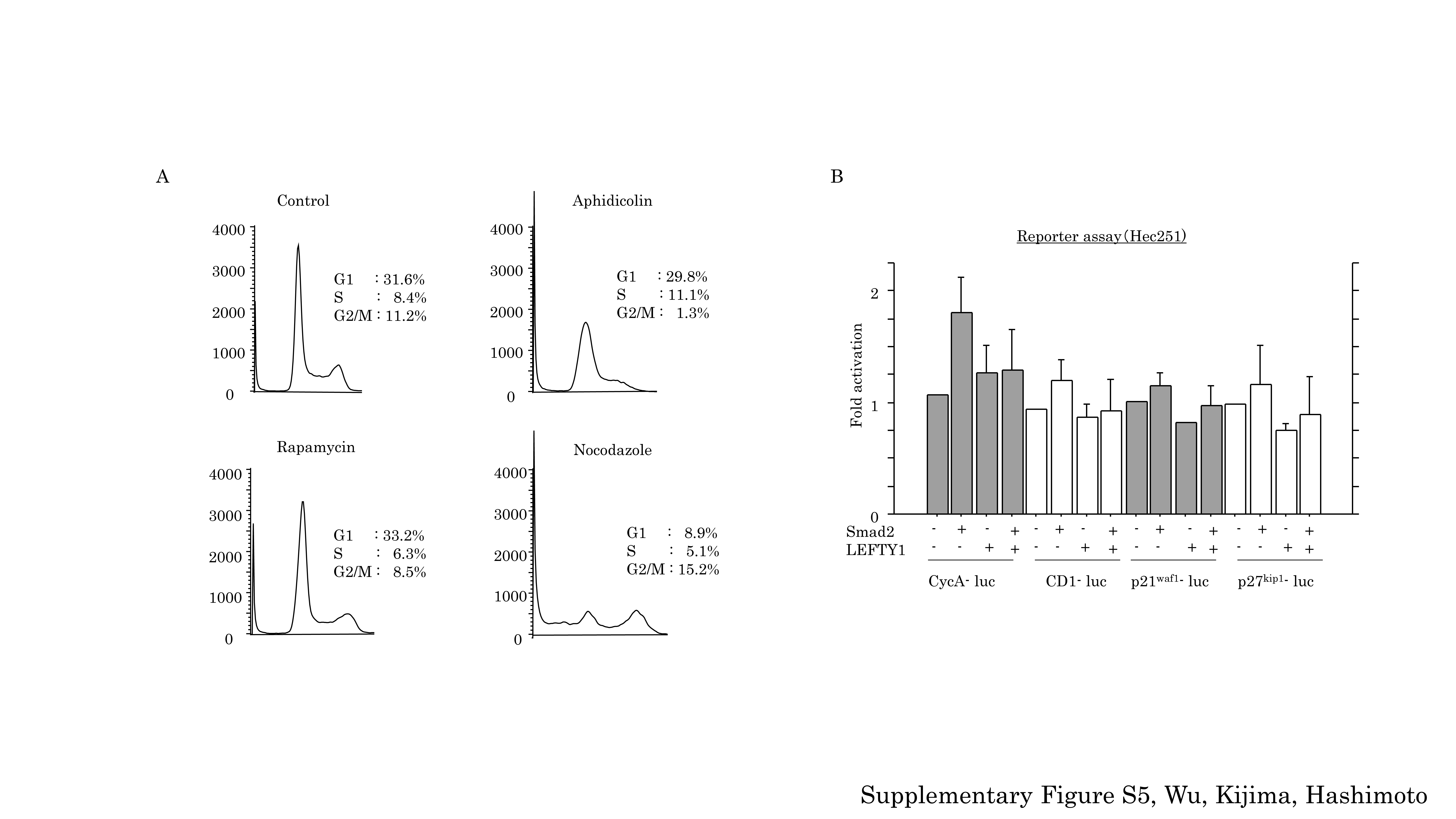

Supplement: Supplementary file 7 — Cell cycle analysis and reporter assay for several cell cycle-related genes. (TIFF 786 kb) [file 12964_2017_211_MOESM7_ESM.tif]

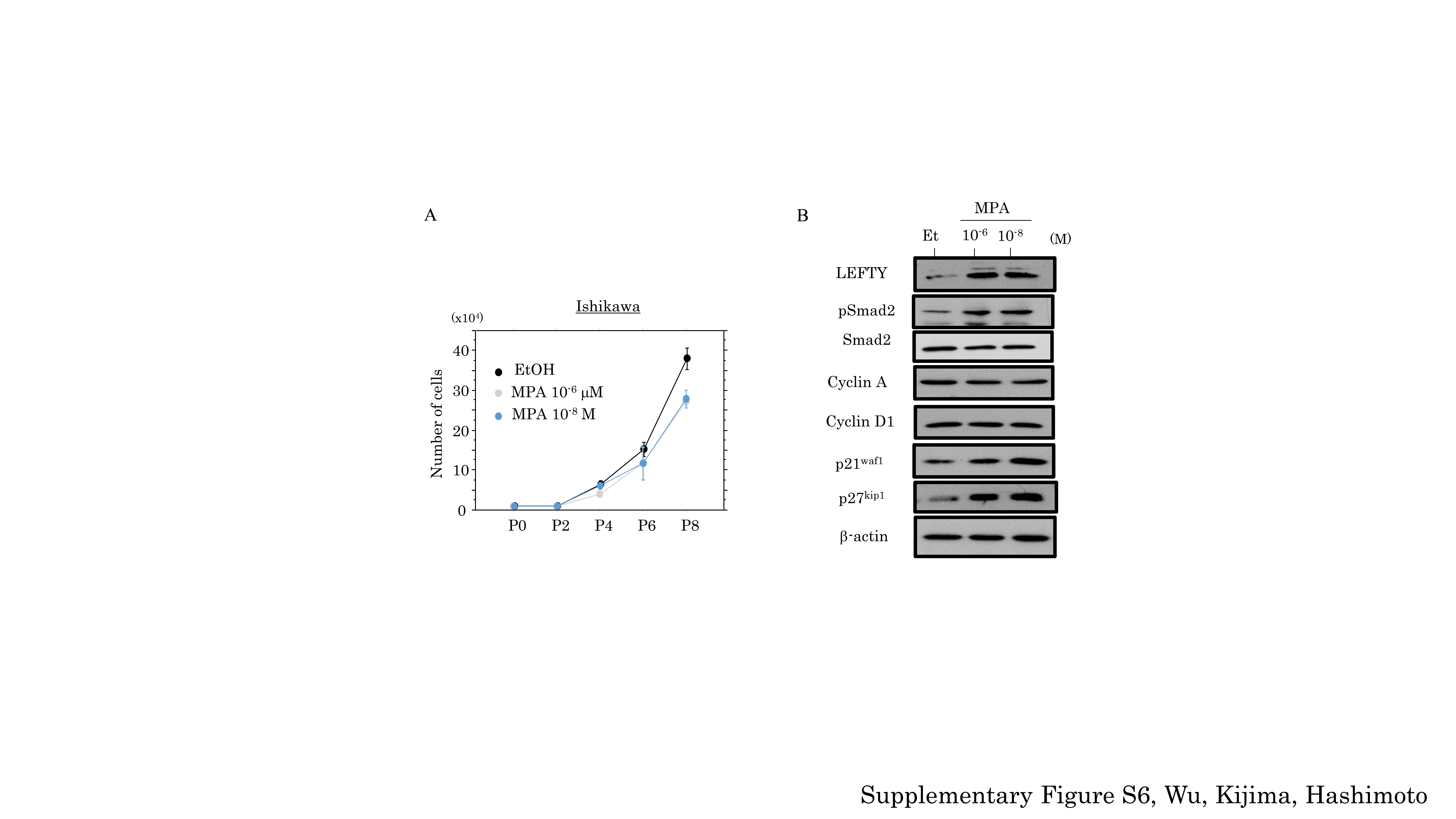

Supplement: Supplementary file 8 — Changes in cell growth and expression of cell cycle-related molecules in Ishikawa cells in response to MPA treatment. (TIFF 1007 kb) [file 12964_2017_211_MOESM8_ESM.tif]

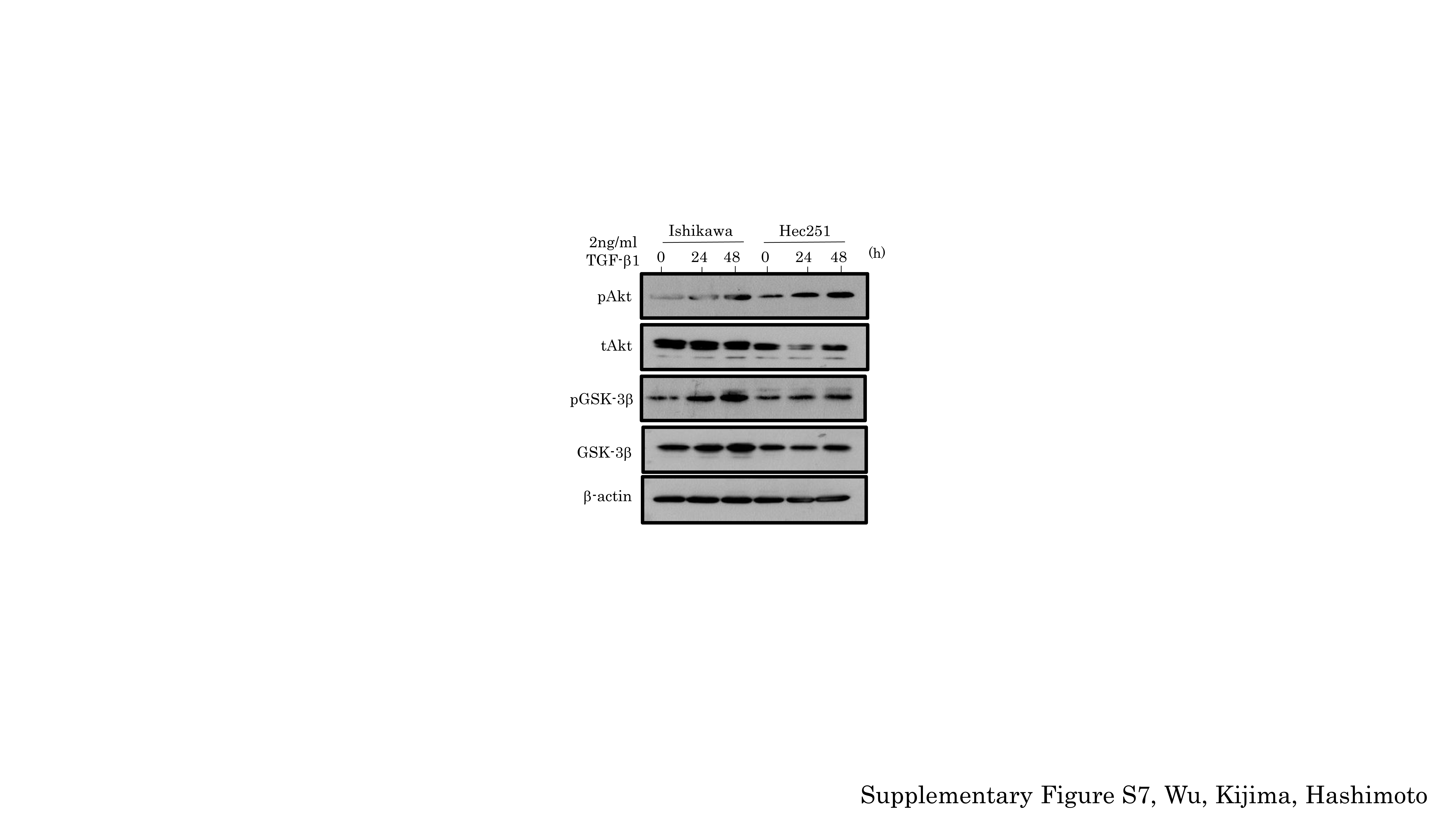

Supplement: Supplementary file 9 — Association between TGF-β and Akt/GSK-3β pathways in endometrial carcinoma cells. (TIFF 1083 kb) [file 12964_2017_211_MOESM9_ESM.tif]
